# Supplementary figures and images for: Regulation of the Yolk Microtubule and Actin Cytoskeleton by Dachsous Cadherins during Zebrafish Epiboly
Source: bioRxiv. 2025 May 14:2025.05.10.653271. Preprint. [Version 1] doi: 10.1101/2025.05.10.653271 (PMC12132318; doi:10.1101/2025.05.10.653271)

## Slide 1
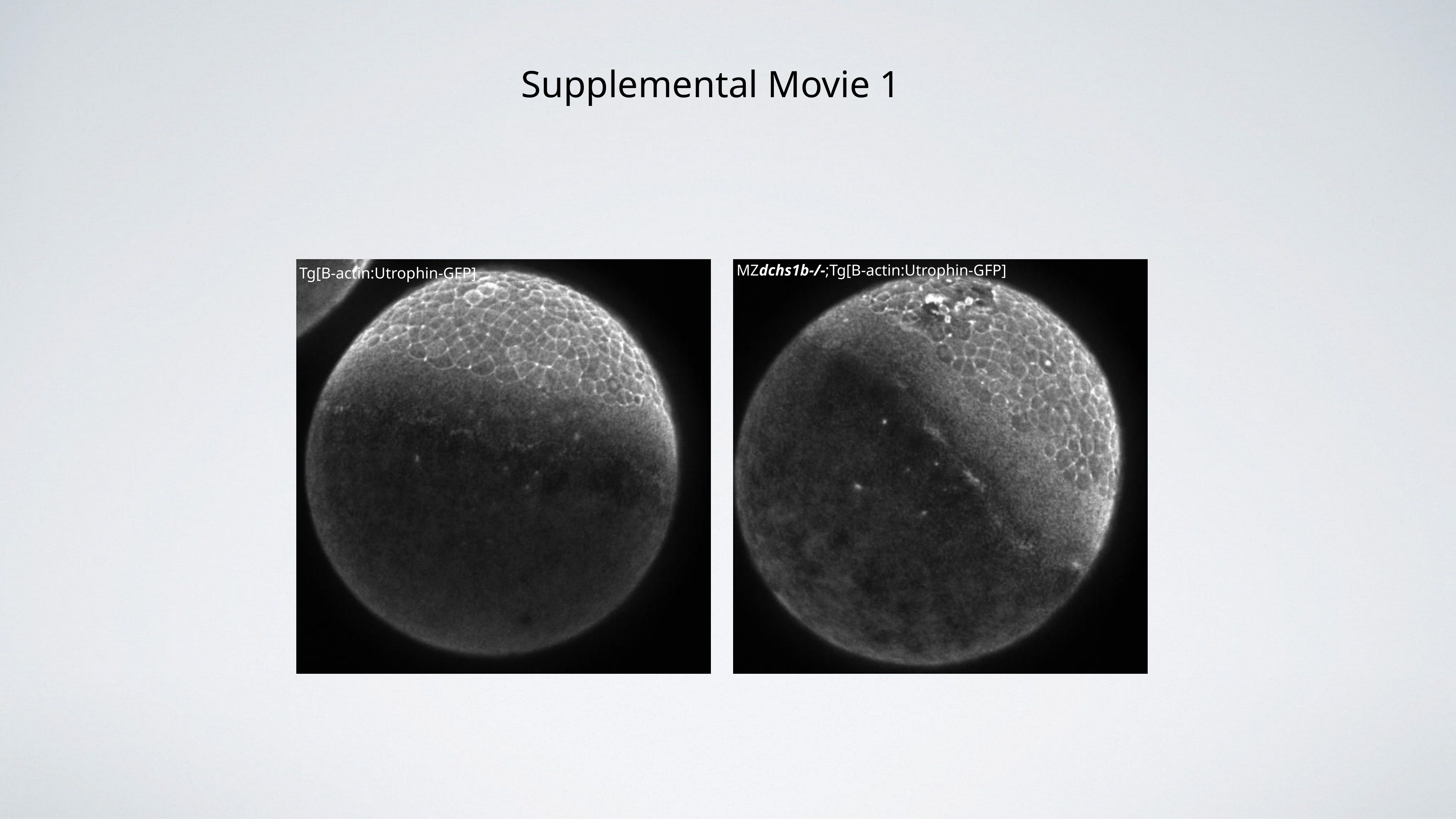

Supplemental Movie 1
MZdchs1b-/-;Tg[B-actin:Utrophin-GFP]
Tg[B-actin:Utrophin-GFP]

Supplement: Supplement 1 [file media-1.pptx]
