# Supplementary material for: Regulation of the Yolk Microtubule and Actin Cytoskeleton by Dachsous Cadherins during Zebrafish Epiboly
Source: bioRxiv. 2025 May 14:2025.05.10.653271. Preprint. [Version 1] doi: 10.1101/2025.05.10.653271 (PMC12132318; doi:10.1101/2025.05.10.653271)

bioRxiv preprint doi: https://doi.org/10.1101/2025.05.10.653271; this version posted May 14, 2025. The copyright holder for this preprint (which was not certified by peer review) is the author/funder, who has granted bioRxiv a license to display the preprint in perpetuity. It is made available under aCC-BY 4.0 International license.

Supplement Figure 1

Additional epiboly progression quantification in wild-type and *dchs* triple mutant embryos, relevant to Figure 1

A. Quantification of maximal YSL-DEL separation during epiboly. Kruskal-Wallis test, multiple comparisons, ns.
B. Quantification of the elapsed time of YSL-DEL separation during epiboly. Kruskal-Wallis test, multiple comparisons, ns.
C. Quantification of elapsed time for DEL closure. Kruskal-Wallis test, multiple comparisons, ns.
D. Quantification of elapsed time for EVL closure. Kruskal-Wallis test, multiple comparisons, ns.

Supplement Figure 2

Expressivity and penetrance of microtubule bundling in compound *dchs* triple mutants, relevant to Figure 3

A. Quantification of yolk microtubule bundling through measurement of bare yolk area lacking microtubules at 50% epiboly. ns (p=0.0887). WT N=25 embryos, MZ*dchs1a*$^{-/-}$; Z*dchs1b*$^{+/-}$; MZ*dchs2*$^{-/-}$ N=29 embryos.
B. Quantification of number of embryos with yolk microtubule bundling. WT N=25 embryos, MZ*dchs1a*$^{-/-}$; Z*dchs1b*$^{+/-}$; MZ*dchs2*$^{-/-}$ N=29 embryos, MZ*dchs1a*$^{-/-}$; Z*dchs1b*$^{-/-}$; MZ*dchs2*$^{-/-}$ N=32 embryos.

Supplement Figure 3

Microtubule polymerization dynamics in MZ*dchs* triple mutants

A. Frequency distribution of EB3 track duration. Error bars indicate SEM. WT N=9 embryos. MZ*dchs1a*$^{-/-}$; MZ*dchs1b*$^{-/-}$; MZ*dchs2*$^{-/-}$ N=14 embryos. Wilcoxon matched-pairs rank test, **p<0.01

bioRxiv preprint doi: https://doi.org/10.1101/2025.05.10.653271; this version posted May 14, 2025. The copyright holder for this preprint (which was not certified by peer review) is the author/funder, who has granted bioRxiv a license to display the preprint in perpetuity. It is made available under aCC-BY 4.0 International license.

B. Frequency distribution of EB3 track average speed. Error bars indicate SEM. WT N=9 embryos. MZ*dchs1a*$^{-/-}$; MZ*dchs1b*$^{-/-}$; MZ*dchs2*$^{-/-}$ N=14 embryos. Wilcoxon matched-pairs rank test, ns
C. Frequency distribution of EB3 track displacement. Error bars indicate SEM. WT N=9 embryos. MZ*dchs1a*$^{-/-}$; MZ*dchs1b*$^{-/-}$; MZ*dchs2*$^{-/-}$ N=14 embryos. Wilcoxon matched-pairs rank test, **p<0.01

Supplement Figure 4

FishEnricher pathway analysis of 781 genes downregulated in MZ*dchs1a,b,2* triple mutants (A-D). FishEnricher pathway analysis of 781 genes upregulated in MZ*dchs1a,b,2* triple mutants (E)

Supplemental Movie 1

Supplemental Movie in support of Figure 7.
Tg(β-actin:utrophin-GFP) in control WT and MZ*dchs1b* mutant embryo imaged in lateral view. In WT the animal pole is at the top and in the mutant, it is tilted to the right. Time-lapse imaging was conducted at 23°C from ~sphere stage, the onset of epiboly, and continued until epiboly was completed in the WT embryo.

bioRxiv preprint doi: https://doi.org/10.1101/2025.05.10.653271; this version posted May 14, 2025. The copyright holder for this preprint (which was not certified by peer review) is the author/funder, who has granted bioRxiv a license to display the preprint in perpetuity. It is made available under aCC-BY 4.0 International license.

Supplement Figure 1

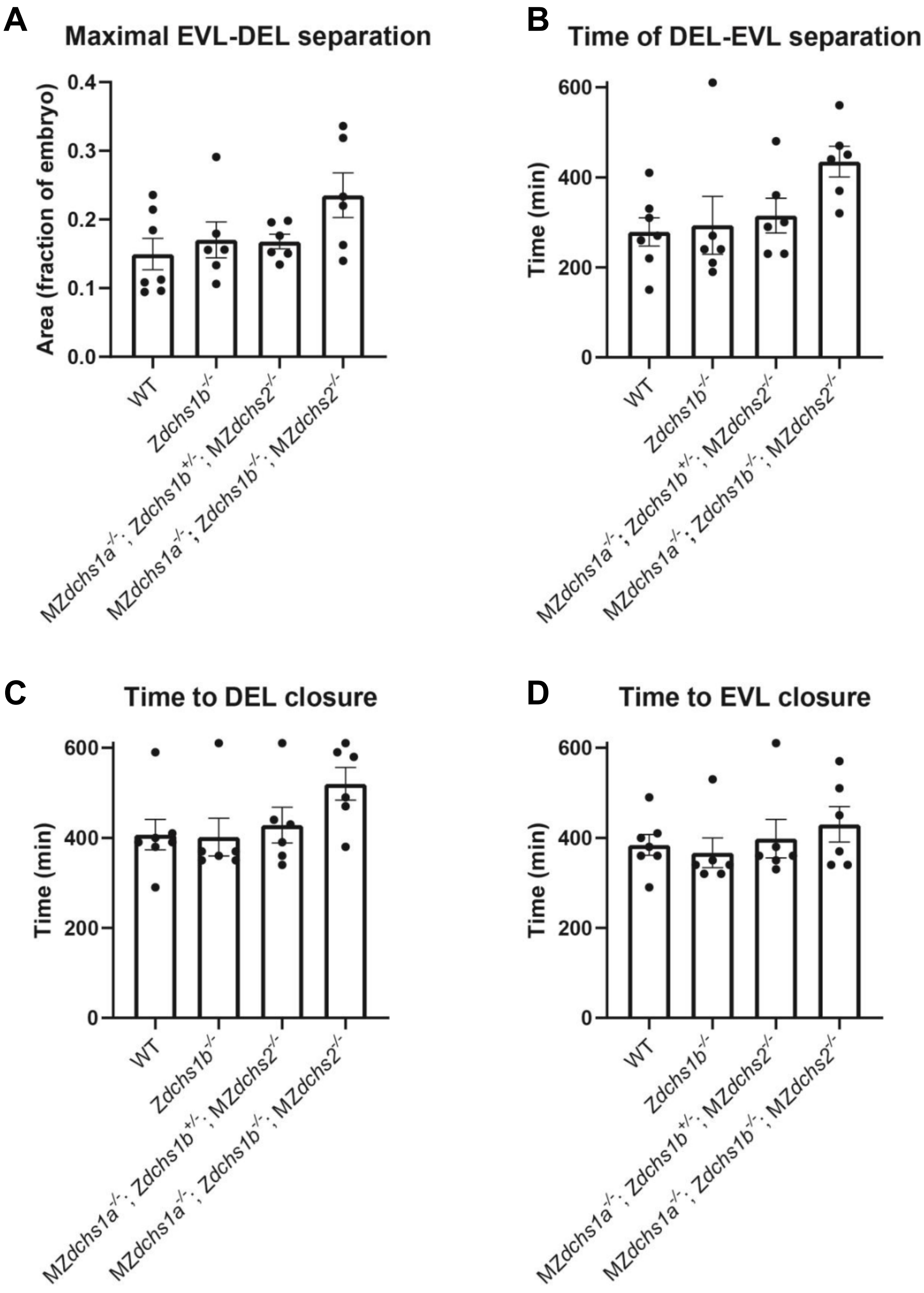

bioRxiv preprint doi: https://doi.org/10.1101/2025.05.10.653271; this version posted May 14, 2025. The copyright holder for this preprint (which was not certified by peer review) is the author/funder, who has granted bioRxiv a license to display the preprint in perpetuity. It is made available under aCC-BY 4.0 International license.

Supplement Figure 2

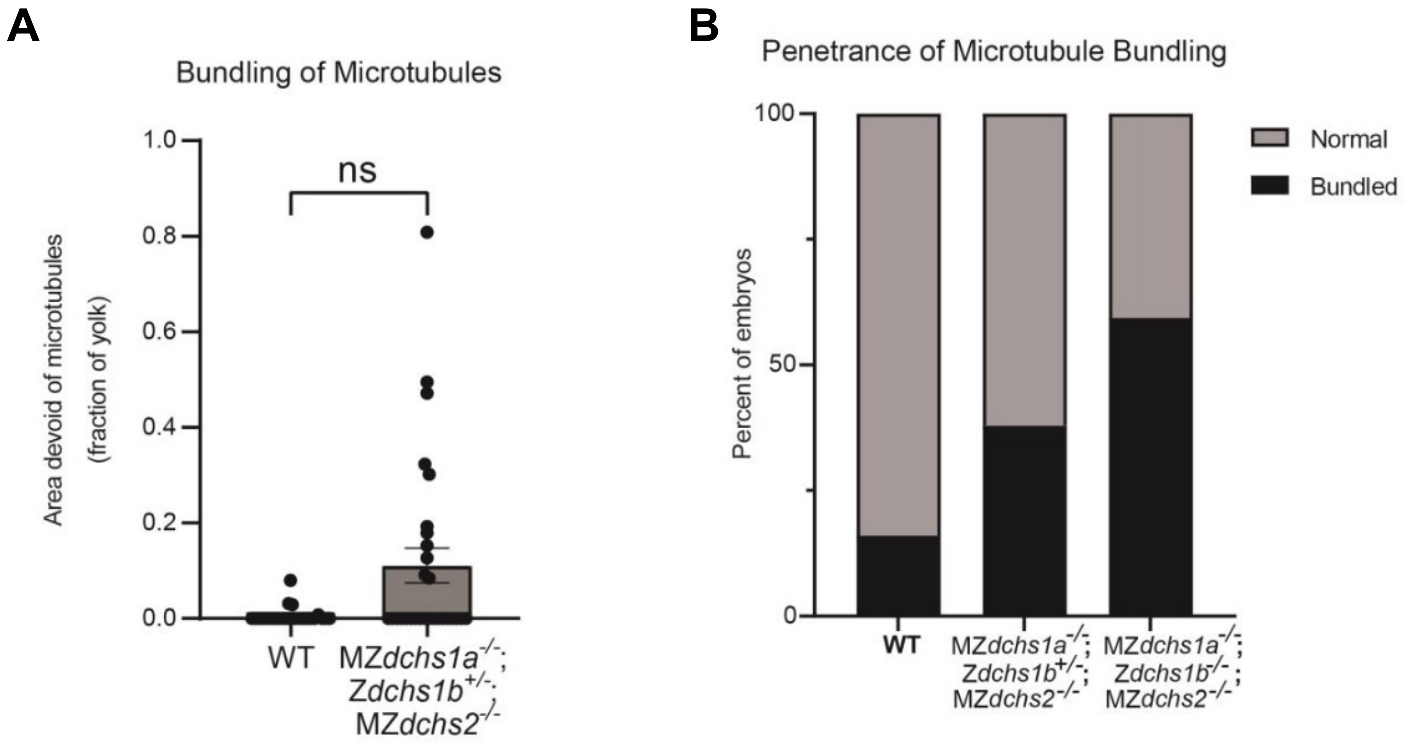

bioRxiv preprint doi: https://doi.org/10.1101/2025.05.10.653271; this version posted May 14, 2025. The copyright holder for this preprint (which was not certified by peer review) is the author/funder, who has granted bioRxiv a license to display the preprint in perpetuity. It is made available under aCC-BY 4.0 International license.

Supplement Figure 3

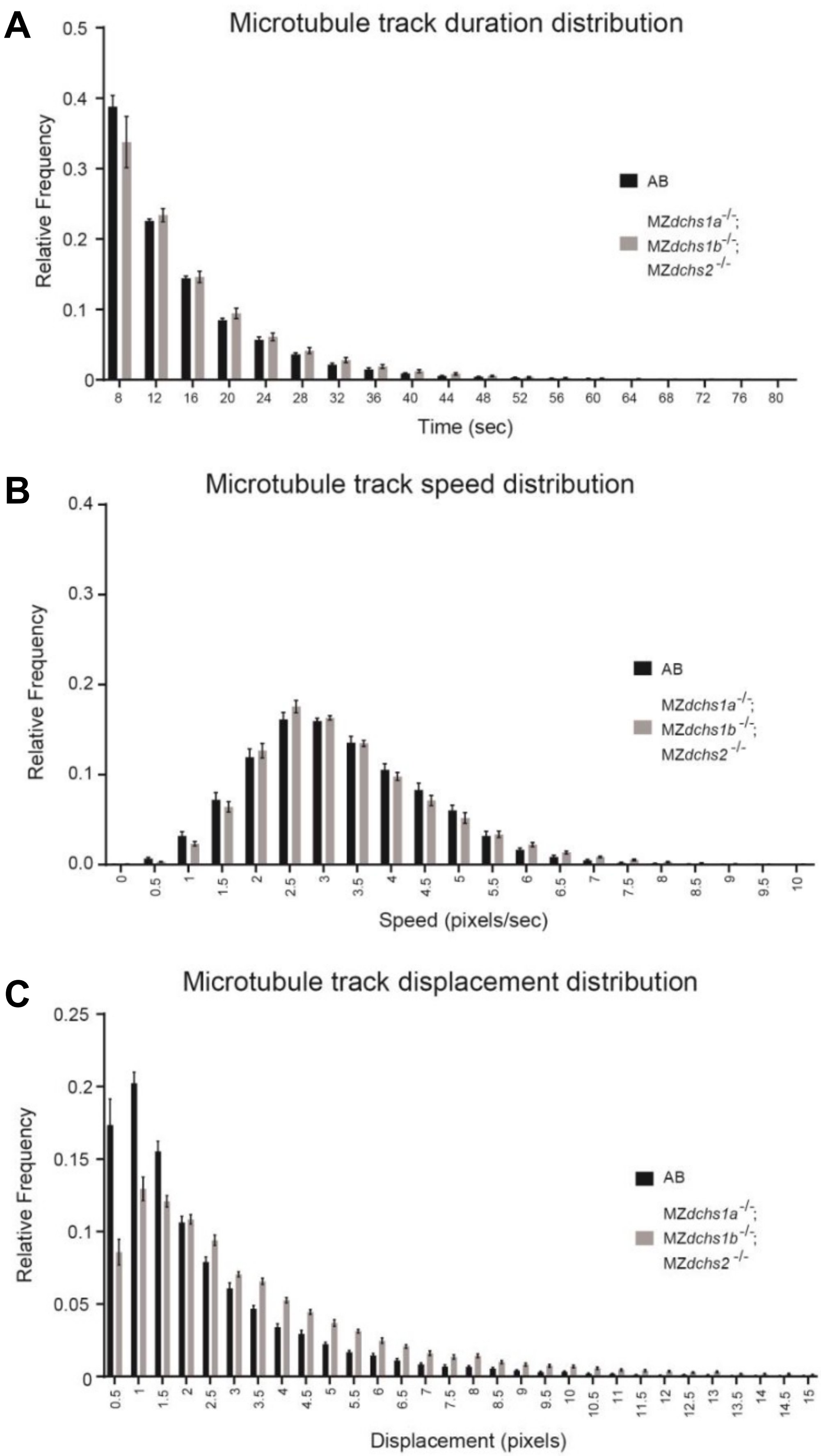

bioRxiv preprint doi: https://doi.org/10.1101/2025.05.10.653271; this version posted May 14, 2025. The copyright holder for this preprint (which was not certified by peer review) is the author/funder, who has granted bioRxiv a license to display the preprint in perpetuity. It is made available under aCC-BY 4.0 International license.

Supplement Figure 4

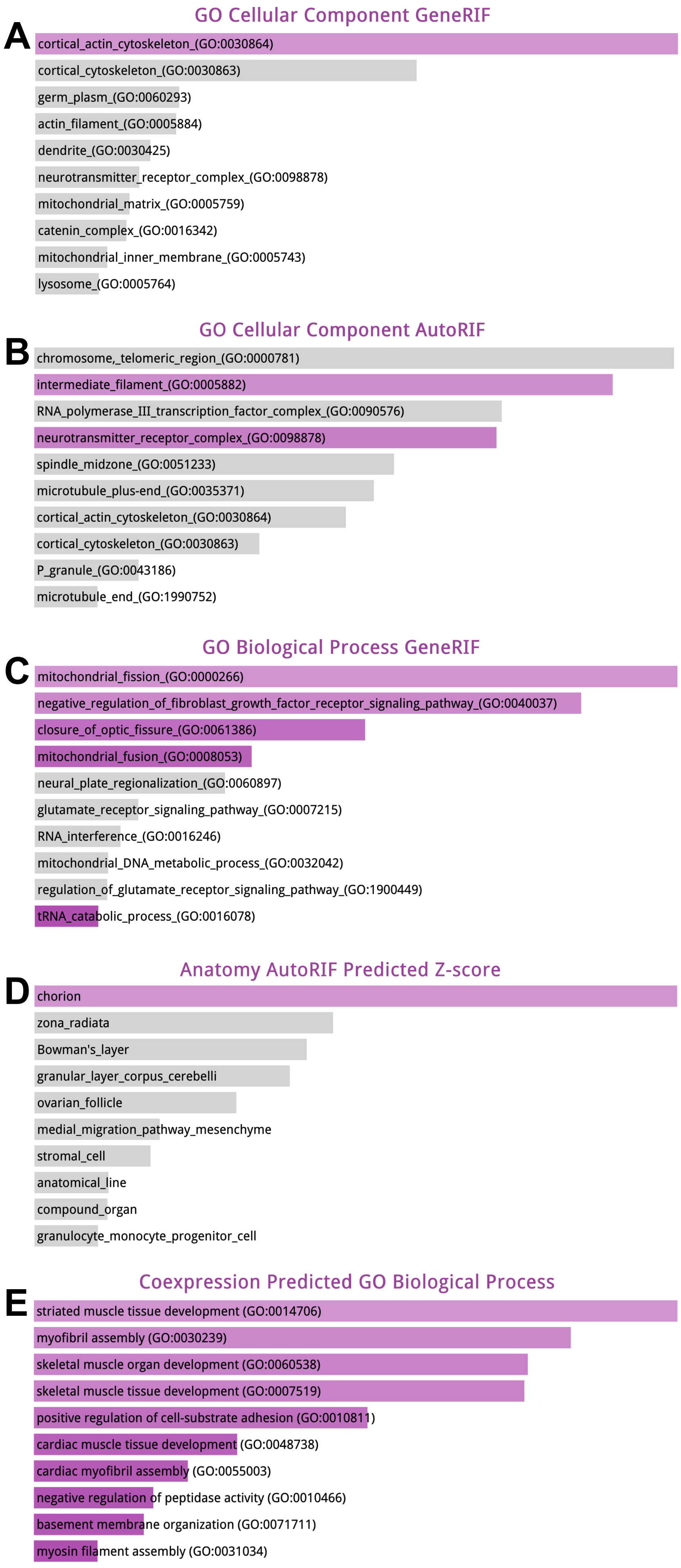

bioRxiv preprint doi: https://doi.org/10.1101/2025.05.10.653271; this version posted May 14, 2025. The copyright holder for this preprint (which was not certified by peer review) is the author/funder, who has granted bioRxiv a license to display the preprint in perpetuity. It is made available under aCC-BY 4.0 International license.

Supplement Movie 1

Tg(β-actin:utrophin-GFP) | **MZ*dchs1b*$^{-/-}$; *Tg(β-actin:utrophin-GFP)***

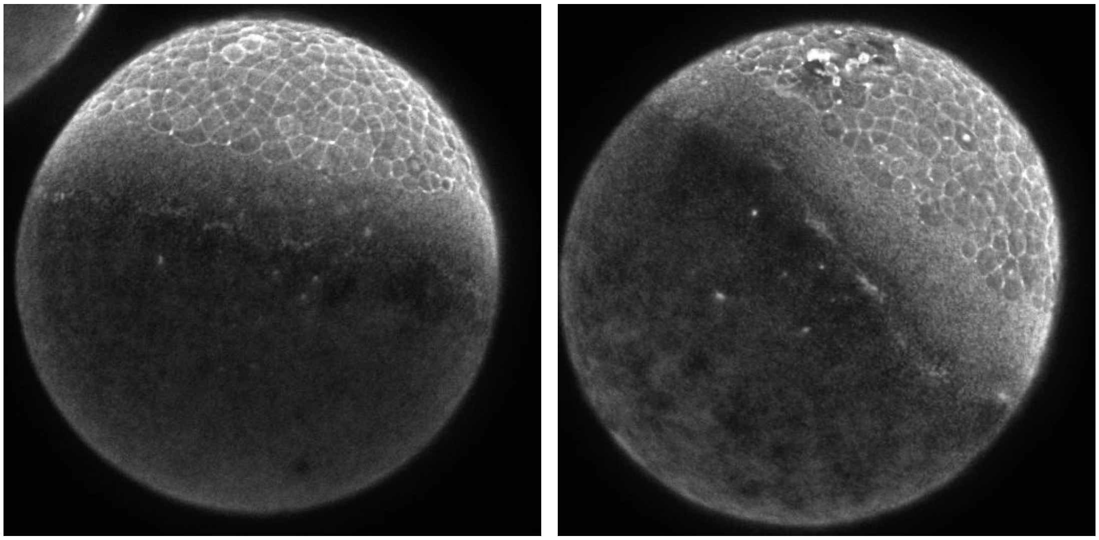

Supplement: Supplement 2 [file NIHPP2025.05.10.653271v1-supplement-2.pdf]
